# Functional Implications of Protein Arginine Methyltransferases (PRMTs) in Neurodegenerative Diseases

**DOI:** 10.3390/biology12091257

**Published:** 2023-09-20

**Authors:** Efthalia Angelopoulou, Efstratios-Stylianos Pyrgelis, Chetana Ahire, Prachi Suman, Awanish Mishra, Christina Piperi

**Affiliations:** 1 1st Department of Neurology, Medical School, National and Kapodistrian University of Athens, Eginition Hospital, 11528 Athens, Greece; angelthal@med.uoa.gr (E.A.); stratospyrg@yahoo.gr (E.-S.P.); 2 Department of Biological Chemistry, Medical School, National and Kapodistrian University of Athens, 11527 Athens, Greece; 3 Department of Pharmacology and Toxicology, National Institute of Pharmaceutical Education and Research (NIPER)-Guwahati, Changsari, Kamrup 781101, Assam, India; chetanaahire512@gmail.com (C.A.); prachigsuman@gmail.com (P.S.)

**Keywords:** arginine methylation, PRMTs, neurodegeneration, Alzheimer’s disease, Parkinson’s disease, amyotrophic lateral sclerosis, Huntington’s disease, spinal muscular atrophy, spinal and bulbar muscular atrophy

## Abstract

**Simple Summary:**

In search of common pathophysiological events among neurodegenerative diseases, arginine methylation of proteins has been revealed as a crucial molecular mechanism regulating several cellular processes, including neuronal cell survival and excitability, axonal transport, synaptic maturation, and myelination. Protein arginine methyltransferases (PRMTs), which catalyze the arginine methylation reaction, are expressed in high levels in the nervous system, and their aberrant function has been recently implicated in the pathophysiological mechanisms underlying several prevalent neurodegenerative diseases, presenting promising future therapeutic targets.

**Abstract:**

During the aging of the global population, the prevalence of neurodegenerative diseases will be continuously growing. Although each disorder is characterized by disease-specific protein accumulations, several common pathophysiological mechanisms encompassing both genetic and environmental factors have been detected. Among them, protein arginine methyltransferases (PRMTs), which catalyze the methylation of arginine of various substrates, have been revealed to regulate several cellular mechanisms, including neuronal cell survival and excitability, axonal transport, synaptic maturation, and myelination. Emerging evidence highlights their critical involvement in the pathophysiology of neurodegenerative diseases, including Alzheimer’s disease (AD), Parkinson’s disease (PD), frontotemporal dementia–amyotrophic lateral sclerosis (FTD-ALS) spectrum, Huntington’s disease (HD), spinal muscular atrophy (SMA) and spinal and bulbar muscular atrophy (SBMA). Underlying mechanisms include the regulation of gene transcription and RNA splicing, as well as their implication in various signaling pathways related to oxidative stress responses, apoptosis, neuroinflammation, vacuole degeneration, abnormal protein accumulation and neurotransmission. The targeting of PRMTs is a therapeutic approach initially developed against various forms of cancer but currently presents a novel potential strategy for neurodegenerative diseases. In this review, we discuss the accumulating evidence on the role of PRMTs in the pathophysiology of neurodegenerative diseases, enlightening their pathogenesis and stimulating future research.

## 1. Introduction

As the global population is getting older, neurodegenerative diseases are becoming a major worldwide health problem. Their clinical presentations vary, with movement disorders and cognitive impairment being the most prevalent. Although each disease is characterized by an accumulation of disease-specific proteins, they all seem to share some common pathophysiological mechanisms, such as oxidative stress, neuroinflammation, ubiquitin–proteasomal and autophagosomal/lysosomal systems impairment, as well as programmed cell death, leading eventually to gradual neuronal dysfunction and death [1,2,3].

Epigenetics involves various heritable changes in gene expression that take place because of environmental effects and do not associate with alterations in the DNA sequence. Neuroepigenetics presents a subdivision of epigenetic mechanisms that affect the nervous system and refers to alterations in neurons that are not propagated to progeny cells since neurons do not divide. The main neuroepigenetic mechanisms involve DNA methylation and hydroxymethylation, histone variants, histone post-translational modifications, miRNAs, lncRNAs and changes in nucleosome positioning and function. An impairment in these systems can unsettle cognitive function and possibly subside to neurodegenerative diseases [4,5,6].

The methylation of arginine residues in proteins can affect crucial signal transduction pathways and cellular functions, compromising gene regulation. It is catalyzed by a specific class of enzymes known as protein arginine methyltransferases (PRMTs) consisting of three main subtypes, I-III, present in mammals and a fourth subtype, IV, present only in yeast. All subtypes catalyze the monomethylation of guanidino nitrogen atoms of arginine residues utilizing the methyl donor S-adenosylmethionine, while Type I and Type II PRMTs can also mediate asymmetric and symmetric dimethylation of the monomethylated arginine residues, respectively [7,8].

PRMTs are highly expressed in the central nervous system (CNS) and have been associated with the development, differentiation and maturation of oligodendrocytes and astrocytes as well as axon myelination [7,9,10], while the lack of some of them has been associated with lethality in animal studies [11]. Furthermore, PRMTs have been associated with neuronal development and function as well as the pathogenesis of several neurodegenerative diseases, including Alzheimer’s and Parkinson’s disease, amyotrophic lateral sclerosis (ALS) and Huntington’s disease (HD) [7,9]. 

Given the significant role of arginine methylation in neurodegeneration and the current lack of comprehensive articles on this important topic, the present review provides a detailed discussion on PRMTs functions and the underlying molecular mechanisms in different neurodegenerative diseases, highlighting the importance of arginine methylation as a potentially promising target for novel therapies.

## 2. Biochemical and Functional Aspects of Arginine Methylation 

Methylation of arginine residues has emerged as a post-translational modification taking place in both histone and non-histone proteins, affecting signaling pathways as well as regulating gene expression. Arginine methylation has been involved in diverse cellular processes such as receptor trafficking, protein–protein interactions, signal transduction, repair of DNA damage, protein stability control and RNA splicing [8]. Methyl group transfer to arginine amino acids is known as N-methylation and is catalyzed by the family of protein arginine-N-methyltransferases (PRMTs), present in the cytoplasm as well as in the cell nucleus. During this reaction, methyl groups are transferred from S-adenosyl-L-methionine (AdoMet) to the guanidino nitrogen atoms of arginine residues, which are present on target proteins, changing their substrate stability, activity, and localization [12,13]. Three different forms of methylarginines have been detected in eukaryotes up to date, including monomethylated arginine (MMA), symmetric dimethylarginine (SDMA) and asymmetric dimethylarginine (ADMA), each produced by a specific PRMT [14]. 

### 2.1. Structural Characteristics of PRMT Family Members 

**Figure 1:**
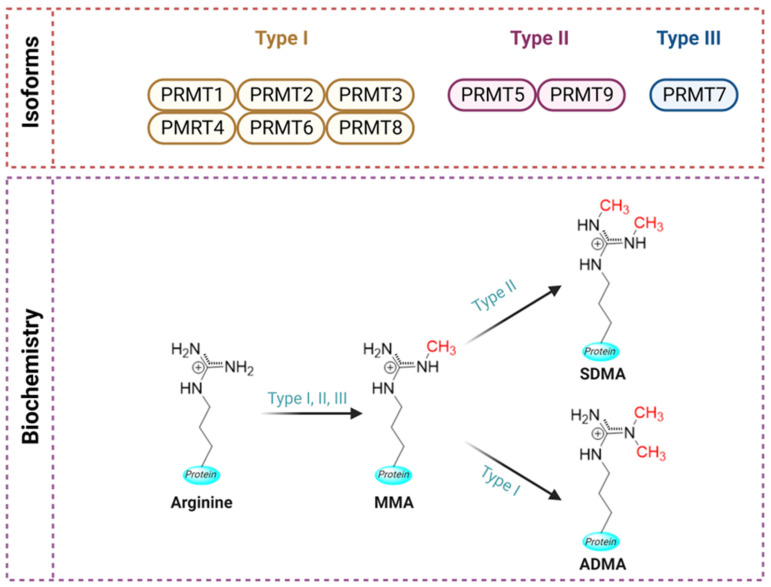
A schematic representation of PRMTs isoforms and their role in arginine methylation. There are three types of PRMTs responsible for conversion of arginine to monomethyl arginine (MMA). Further generation of symmetrical dimethylarginine (SDMA) is catalyzed by type II PMRTs, while formation of asymmetrical dimethylarginine (ADMA) is catalyzed by type I PMRTs (Created with BioRender.com, accessed on 11 August 2023).

All members of the PRMT family are class I enzymes containing four characteristic motifs (namely motif I, post-I, II, and III) as well as a conserved THW loop [15]. The formation of the AdoMet-binding pocket requires the presence of motifs I and post-I, as well as the THW loop [16]. Most PRMTs can methylate glycine- and arginine-rich patches (known GAR motifs) in respective substrates except for the coactivator-associated arginine methyltransferase 1 (CARM1 or PRMT4). Based on their catalytic activity, the PRMT family is composed of four types of enzymes (Types I-IV) [7]. The different isoforms of PRMTs and their role in the methylation of arginine have been illustrated in Figure 1.

Type I PRMTs are the most common, inducing asymmetric dimethylation by transferring two methyl groups to the terminal nitrogen atoms of arginines (ω-N^G^, NG-dimethylarginine). They consist of six enzymes: PRMT1-4, PRMT6, and PRMT8, with PRMT1 being the most predominant type [14]. 

Type II PRMTs mediate the symmetric dimethylation of arginines by transferring one methyl group to the terminal nitrogen atoms of arginines [8,17]. PRMT5 and PRMT9 belong to Type II PRMTs [18], with PRMT9 being recently shown to methylate the RNA splicing factor SF3B2 [19,20]. 

Type III PRMT (PRMT7) catalyzes monomethylation of arginines as its final reaction product [21], while Type IV enzymes mediate arginine monomethylation of internal guanidino nitrogen only in yeast, without any mammalian homologs present [22].

Although most PRMTs are active upon expression, several mechanisms have been detected to regulate their activity, including interaction with regulatory proteins, post-translational modifications, control of RNA levels by micro-RNAs and subcellular compartmentalization. More specifically, phosphorylation and glycosylation of PRMT4 (CARM1) have an activating role on the enzyme [23,24], while phosphorylation of PRMT5 inactivates it by blocking its interaction with methylosome protein 50 (MEP50) [25]. Moreover, MEP50 and the SWI/SNF chromatin complex were shown to be required for PRMT5 activation [26]. Additionally, the interaction of BTG1-binding chromatin assembly factor 1 (CAF1) with PRMT1 has a negative impact on its activity [27], while the CCCTC-binding factor like (CTCFL) enhances PRMT7 activity [28]. Regarding regulation of PRMTs by miRNAs, PRMT5 has been demonstrated to be regulated by miR-92, miR-96, miR-25, and miR-32 [29].

### 2.2. CNS Functions of PRMT Family Members

Most PRMTs are expressed in high levels and function in the CNS (Table 1) [30,31]. PRMT1 has been essential for early brain development and has been shown to be required for the formation of astrocytes, oligodendrocytes, and neurons [10]. Conditional deletion of PRMT1 in murine neural stem cells was demonstrated to lead to neonatal death due to severe myelination defects of neurons [11]. Moreover, PRMT1 regulates positively the activity of KCNQ ion channels, which are critical for neuronal excitability [32]. PRMT3, on the other hand, has been demonstrated to be required for dendritic spine maturation in rat hippocampal neurons [33]. Additionally, PRMT4 has been involved in the proliferative state of neural progenitor cells through methylation of the RNA binding protein HuD, blocking its binding to the cyclin-dependent kinase inhibitor, p21cip1/waf1 [34]. PRMT4 can also regulate astroglial cell fate by methylating histone H3 arginine 17 [35], while PRMT6 has been recently shown to affect axonal transport and neuronal viability [36]. PRMT8 can control the maturation of the synapses, and loss of PRMT8 was shown to affect hippocampus-dependent memory [37,38].

Type II PRMT5 has been implicated in the differentiation of oligodendrocytes and in the myelination process [11,39], while experiments of PRMT5 deletion in neural stem cells led to neonatal death [40]. Moreover, PRMT5 was shown to participate in neural stem cell proliferation through the symmetric dimethylation of histone H4 arginine 3 [41].

Type III PRMT7 has been shown to regulate sodium leak channel NALCN activity, which establishes the resting membrane potential (RMP) in neurons and increases neuronal excitability through the regulation of extracellular Ca^2+^ levels [42]. 

**Table 1:** PRMT classification and CNS functions.

PRMT Family Member	Enzyme Type	Methylation Reaction	CNS Function	Ref.
PRMT1	I	asymmetric dimethylation	- development of astrocytes, oligodendrocytes and neurons	[10]
			- neurons myelination	[11]
			- positive regulation of KCNQ ion channel activity and neuron excitability	[32]
PRMT2	I	asymmetric dimethylation		
PRMT3	I	asymmetric dimethylation	- essential for dendritic spine maturation in rat hippocampal neurons	[33]
PRMT4 (CARM1)	I	asymmetric dimethylation	- maintenance of neural progenitor cell proliferation	[34]
			- regulation of astroglial cell fate	[35]
PRMT5	II	symmetric dimethylation	- differentiation of oligodendrocytes and myelination process	[11,39]
			- neural stem cell proliferation	[41]
PRMT6	I	asymmetric dimethylation	- affects axonal transport and neuronal viability	[36]
PRMT7	III	arginine monomethylation	- regulation of sodium leak NALCN ion channel activity and neuron excitability	[42]
PRMT8	I	asymmetric dimethylation	- control of the maturation of synapses	[37]
			- affects hippocampus-dependent memory	[38]
PRMT9	II	symmetric dimethylation		[18]

## 3. Role of PRMTs in Alzheimer’s Disease

Alzheimer’s disease (AD) is the most prevalent neurodegenerative disorder, which is mainly denoted by cognitive impairment. Senile plaques (SPs) and Neurofibrillary tangles (NFTs) were once thought to be the two main characteristics of AD [43,44]. However, it has now been discovered that genetic and epigenetic mechanisms also play a significant role in the disease’s pathophysiology. Recent studies have demonstrated that PRMTs (and specifically PRMT4, PRMT5 and PRMT8) are implicated in the disease and are involved in the modulation of various signaling pathways, which eventually cause accumulation of beta-amyloid, tau phosphorylation, neuroinflammation and cell death [45].

### 3.1. PRMT4 in Nitric Oxide Dysregulation

**Figure 2:**
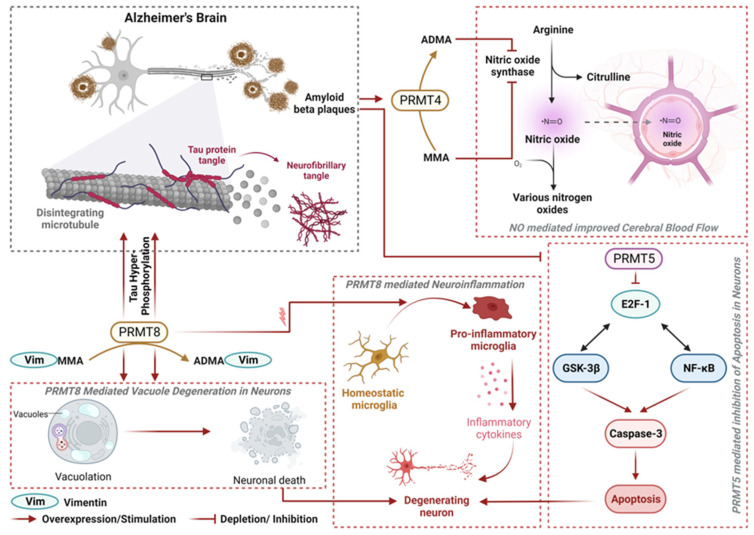
Intricated role of PRMTs in Alzheimer’s Disease pathology. ADMA formation via PRMT4 binds to nitric oxide synthase (NOS) as a non-canonical ligand and inhibits NOS function while reducing cerebral blood flow, an important parameter for normal brain functions. Accumulation of amyloid beta leads to depletion of PRMT5 and potentiates neuronal apoptosis, further enhancing neuronal degeneration. Overexpression of PRMT8 leads to tau hyperphosphorylation, neuroinflammation and vacuole degeneration in neurons (Created with BioRender.com, accessed on 11 August 2023).

There is evidence that the dysregulation of cerebral blood flow (CBF) in AD is related to the loss of NO signaling, a vasodilatory gasotransmitter [46]. Nitric oxide synthase (NOS), a member of the NOS family of enzymes that primarily consists of endothelial NOS (eNOS), neuronal NOS (nNOS), and inducible NOS (iNOS), produces NO. It has been shown that NO levels are lower in AD. NO formation requires the binding of NOS to arginine, its canonical ligand. However, asymmetric dimethylarginine (ADMA) can also bind to NOS as a non-canonical ligand, inhibiting NOS uncoupling, which denotes an incompatibility between NOS function and NO formation (elaborated in Figure 2). As previously described, Type I PRMT activity is the main source of ADMA in our bodies, while symmetric dimethylarginine (SDMA) and mono methylarginine (MMA) are formed by Type II and Type III PRMTs, respectively. When ADMA binds to eNOS, the auxiliary heme group of the enzyme increases in spin state, which weakens the stability of electron transport. This faulty electron transfer has been suggested to be a contributing factor in the development of free radical species, such as peroxynitrite (ONOO−). This uncoupling process has been linked to aging in AD and in other age-related pathologies [46]. NO levels drop because of the conversion of NO to ONOO−, which in turn affects NO’s capacity to control CBF. Vasoregulation gets disrupted inside the brain, which has serious consequences, such as tissue damage and neurocognitive deterioration because of inadequate blood flow. These tissue-damaging effects eventually cause the ventricles to enlarge, which is seen in AD pathology. It is also elucidated that the formation of SPs and NFTs is related to decreased eNOS expression in capillaries [47]. The vascular microenvironment needs to be stabilized and regulated, and eNOS-derived NO is essential for this. Numerous pieces of evidence point to the possibility that eNOS-derived NO can directly regulate the production of Aβ and guard against an increase in its level [48]. But since NO is a free radical by nature, especially if it comes from inducible NOS (iNOS), its excess can cause neurotoxicity and neurodegeneration.

### 3.2. PRMT5 in Amyloidosis

PRMT5 catalyzes the mono- and symmetric dimethylation of arginine residues in proteins, which regulate cell development and death. PRMT5 is mostly expressed in human nerve cells, and in normal conditions, it inhibits the apoptosis process by downregulating the E2F-1/p53/Bax/NF-κB/GSK-3β/Caspase 3 signaling pathway [49]. In pathological conditions, the accumulation of amyloid beta results in the inhibition of PRMT5 [46], which further potentiates the apoptosis process (Figure 2).

A buildup of toxic Aβ, aberrant Tau modifications, and gene mutations have been described as potential AD pathophysiological mechanisms [50]. GSK-3β has been demonstrated to alter the function of these proteins through the phosphorylation of particular amino acid hydroxyl groups in its downstream substrate protein, contributing to cell death and the onset of neurological disorders. An essential multipurpose transcription factor in microglia, NF-κB, is implicated in their activation and can cause inflammation-induced brain damage. According to studies, excessive stimulation of NF-κB can cause neurological deterioration, and aberrant NF-κB expression has been seen in the damage sites of neurodegenerative disorders [51]. Another crucial transcription factor is E2F-1, which can trigger the expression of specific genes and prompt cell cycle changes. Evidence supports the notion that PRMT5 controls the growth, maintenance, and regeneration of both muscle and neurons. IκB degradation and NF-κB activation were linked to PRMT5 depletion [52]. Induction of Aβ in primary cortical neurons leads to a 54% drop in PRMT5 expression and a 70% increase in E2F-1 expression. Studies have revealed that the protein Tau in the brain tissues of AD patients has detectable phosphorylation changes at Thr231, Ser235, Ser396, Tyr394, Ser404, and Thr181 [53], suggesting that the protein is intimately linked to neurodegeneration. Further, activation of GSK-3β results in the phosphorylation of Tau at the Ser404 location and leads to the development of AD [54]. Research data show a link between GSK-3β and the death of nerve cells, the atrophy of neuron protrusions, and cognitive decline. However, the exact mechanism behind this event is still not clear.

### 3.3. PRMT8 in Vacuole Degeneration 

PRMT8 is most prominently expressed in the CNS, and its overexpression has been shown to enhance neuroinflammation, vacuole degeneration, and tau phosphorylation. Additionally, proteomic studies revealed that overexpression of PRMT8 induces arginine methylation of vimentin, a type III intermediate filament (IF) protein that is essential for cytosolic organelle placement [55]. Although astrocytes in the brain express vimentin primarily, it is also expressed in neuronal cells and localizes to the perikarya and dendrites. Vimentin has been shown to play a role in the dendritic damage response pathway in an AD mouse model [56], and vimentin’s arginine methylation by PRMT8 may, therefore, contribute to dendritic cell harm. Studies have shown that the brain tissue of mice overexpressing PRMT8 has a distinctive lesion. There were many HE-negative vacuoles found in the hippocampus regions where PRMT8 was overexpressed [57]. Additionally, there was a rise in cleaved caspase-3 levels in the vicinity of these vacuoles, indicating that vacuolization under these circumstances might be connected to cell death (elaborated in Figure 2). Most of the time, cellular components, including synaptic terminals and dendrites, swell to generate the vacuoles. In PRMT8-overexpressing animals, it was observed that there is reduced expression of the dendritic marker microtubule-associated protein 2 (MAP-2), which is consistent with PRMT8 overexpression causing dendrite structural disruption. In addition, PRMT8 overexpression results in activation of microglia [55]. These reactive microglia secrete pro-inflammatory cytokines, leading to neuroinflammation (elaborated in Figure 2). Overall, the effect of amyloid beta on PRMT8 expression is still unclear. 

In summary, ADMA formation via PRMT4 can bind to NOS as a non-canonical ligand and inhibit its function while decreasing cerebral blood flow. Accumulation of amyloid beta can also result in PRMT5 depletion; hence, reduction in PRMT5-mediated inhibition of apoptosis in neurons may further potentiate neuronal degeneration. PRMT8 overexpression may result in tau hyperphosphorylation, neuroinflammation and vacuole degeneration in neurons.

## 4. Role of PRMTs in the Spectrum of Frontotemporal Dementia (FTD)—Amyotrophic Lateral Sclerosis (ALS)

Frontotemporal dementia (FTD) is a type of neurodegenerative dementia denoted by the loss of neurons selectively in the frontal and temporal lobes that causes personality and social behavioral problems, semantic dementia, and progressive non-fluent aphasia. Amyotrophic lateral Sclerosis (ALS) is a neurodegenerative disease determined by the loss of upper and lower motor neurons, resulting in progressive muscle weakness, atrophy, and spasticity. FTD and ALS are two neurodegenerative disorders that share some pathogenic genes, such as Chromosome 9 open reading frame 72 (*C9ORF72)*, TAR DNA-binding protein (*TARDBP)* and fused in sarcoma, *FUS*. Moreover, some patients with ALS present symptoms of FTD and *vice versa*. Therefore, we conclude that these two diseases may share some pathological mechanisms [58]. The mean survival of FTD ranges between 8 and 12 years, depending on the specific phenotype, while in ALS, mean survival is 1–5 years, with most patients dying due to respiratory paralysis [59,60,61,62]. 

The majority of ALS cases are sporadic. However, in familial cases, which account for 10%, genetic components are important [59]. One established genetic cause of ALS and FTD is the repeat-expansion mutation in the *C9orf72* gene [57]. Many other causative mutations have been detected in familial ALS, such as superoxide dismutase1 (SOD1) mutations in core genes such as *TARDBP* and *FUS* [54]. 

In cases with repeat-expansions in *C9orf72,* which is responsible for approximately 40% of familial and 5% of sporadic ALS cases, loss of function and gain-of-function toxicity have been observed. This cytotoxicity concerns arginine-rich dipeptide repeat proteins (DRPs) called polyGR and polyPR. It has been shown that inhibition of Type I protein arginine methyltransferases (PRMT1) protects against this toxicity in motor neurons in mice. Importantly, according to one study, the inhibition of asymmetric arginine dimethylation by PRMT1 inhibitor MS023 may protect against toxicity, rescuing neuron survival and preserving neurite integrity. Various roles of protein arginine methylation as a response to cell stress have also been described, such as chromatin remodeling, splicing, and stress granule dynamics in *C9orf72* mutations. A possible association between the C9orf72 protein and PRMTs in regulating pathways in neurodegenerative disorders has been detected [61]. Moreover, PRMTs may regulate autophagy [61]. This is important, as symmetric dimethylation of stress granule proteins by PRMT5 has been found to reduce stress granules by autophagy [63]. 

The mutation in the FUS protein accounts for 5% of familial ALS. FUS is a DNA/RNA binding protein, which belongs to a group of RBPs called FET (FUS, EWSR1, and TAF15) and includes Ewing’s sarcoma RNA binding protein 1 (EWSR1) and TATA-binding protein associated factor 2N [64]. Various mutations in *FUS* are known to be associated with familial ALS, and through pathologic mechanisms, these mutations change protein–protein and protein–RNA complexes leading to neurodegeneration [59]. FUS plays a very important role in various cellular processes, such as RNA transport, mRNA stability, synaptic homeostasis, RNA splicing, DNA repair and damage response [60,64]. The control of FUS localization is of great importance since mutations that cause delocalization of intracellular FUS and FUS inclusions are associated with ALS [60,64]. Apart from ALS, FTD has also been associated with inclusions containing FUS, even though these mutations are found in less than 1% of FTD cases [58,59]. In concrete subtypes of ALS and frontotemporal lobar degeneration (FTLD), called ALS-FUS and FTLD-FUS, cytoplasmic and nuclear inclusions of FUS are the characteristic features. The majority of FUS mutations are in the proline–tyrosine nuclear localization sequence of the C-terminal. Methylation of FUS by PRMTs reduced transport binding and led to impairment of nuclear translocation of FUS. 

PRMT1 promotes arginine methylation that affects the localization of nuclear ribonucleoproteins (RBPs), such as FUS RBP [64]. PRMT1 is responsible for many processes, including signal transduction, DNA repair, cellular localization, and transcriptional regulation. This PRMT causes most of the cellular methylation. PRMT1 is located in the nucleus and cytosol and is able to translocate between the two places. Arginine methylation by PRMT1 controls stress granule (SG) assembly, while localization of FUS relies on cell types and stress conditions. The firm complex of FUS-R521C with PRMT1 leads to SG aggregates and neurite morphology disturbance and can possibly contribute to neurodegeneration [59]. PRMT1 regulates neuronal cytoplasmic shuttling of FUS. In ALS, asymmetric methylation of FUS catalyzed by PRMTs can lead to the formation of cytoplasmic inclusions. Specifically, in ALS wild type and mutant FUS cause loss of nuclear PRMT1 function, which is correlated with disturbance of localization of FUS in the cytoplasm, as the loss of nuclear PRMT1 is associated with cytoplasmic mislocalization of FUS. Under normal circumstances, PRMT1 transports between nucleus and cytoplasm. However, loss of nuclear PRMT1 leads to abnormal cytoplasmic FUS accumulation, decreasing transcriptional neuronal activity [60]. PRMT1 is sequestered into cytosolic SG aggregates and is a crucial regulator of FUS intracellular localization and formation of FUS SGs. Thus, PRMT1 was observed to affect the localization of FUS (nuclear or cytoplasmatic), formation of SG, and cellular toxicity due to FUS mutants, being associated with ALS-linked FUS-R521C [59,64]. The aggregation of FUS requires processes, some of which are RNA-dependent and some RNA-independent [59]. 

During oxidative stress, FUS-R521C, along with PRMT1, tends to be aggregated to cytosolic SG. Oxidative stress-induced overexpression or PRMT1 loss regulates cytosolic FUS aggregates and cortical neuron morphology. In such conditions, manipulation of PRMT1 by overexpression and down-regulation of FUS-R521C expressing neurons also controls neurite morphology during oxidative stress. The dysregulation of Nd1-L mRNA, an actin-stabilizing protein, is an established RNA target of FUS in post-mitotic neurons and affects their morphology and function. More specifically, expression of Nd1-L improves neurite shortening and loss of Nd1-L aggravates neurite shortening. Nd1-L mRNAs create FUS aggregates, which are gathered into FUS-R521C/PRMT1 complexes. Different ALS-associated FUS mutants change protein–protein and protein–RNA interactions, contributing to neurite degeneration upon oxidative stress. It has been demonstrated that both PRMT1 and Nd1-L are strongly associated with ALS-linked FUS mutants. Moreover, PRMT1 is associated more with FUS-R521C than with FUS-WT or FUS-P525L, driving to insufficient arginine methylation of ubiquitin-associated protein 2-like (UBAP2L), leading to FUS-R521C-positive SGs staying in the cytoplasm. This mutant is frequently localized to SGs, which leads to FUS-positive SG aggregates. The scarce FUS-P525L mutation causes a more severe form of ALS in young patients. This rare mutation is localized in the cytosol and forms cytosolic aggregates, whereas the FUS-R521C mutation is localized in both the cytosol and the nucleus, forming aggregates, respectively [59].

RALY is an RNA-binding protein of a group of nuclear ribonucleoproteins that interacts with PRMT1 mRNA, the downregulation of which causes decreased levels of PRMT1 mRNA and protein. The expression of PRMT1 has been found enriched in RALY-containing RNPs, while on RALY-silencing, this protein has been found to be downregulated. Thus, RALY downregulation reduces levels of PRMT1 and, thus, FUS methylation, restoring the nuclear translocation of ALS-linked FUS mutants. FUS mutations lead to cytosolic aggregate formation that is associated with ALS [64].

In conclusion, FTD and ALS, in many cases, coexist and share some pathological mechanisms and some causative gene mutations, among which are expansions in the *C9orf72* gene and mutations in the FUS protein. The last one is a ribonucleoprotein that regulates FUS methylation and, thus, its cytosolic localizations. Loss of PRMT1 accumulates FUS-positive aggregates and contributes to neurite degeneration, whereas overexpression of PRMT1 protects from this degeneration. New insights into the role of PRMTs could lead to novel treatment alternatives for severe and irreversible neurodegenerative disorders, such as FTD and ALS.

## 5. Role of PRMTs in Parkinson’s Disease

Parkinson’s disease (PD) is the second most prevalent neurodegenerative disorder after AD, clinically determined by rigidity, resting tremor, bradykinesia, postural instability, and diverse non-motor manifestations, including sleep disturbances, depression, psychotic features, cognitive impairment, and autonomic dysfunction [65]. The onset age of PD is usually between 55 and 65 years [66]. The mean duration of the disease is ten years, although the rate of progression is highly variable. Older age at onset has been associated with more rapid progression and cognitive impairment [66], while patients with an earlier onset display longer survival, delayed onset of falls, but more commonly levodopa-induced motor complications [67,68]. Key neuropathological features of PD are dopaminergic neuronal death in the substantia nigra pars compacta (SNpc), subsequent nigrostriatal denervation, and the accumulation of Lewy bodies and Lewy neurites in the remaining neurons consisting of α-synuclein among other proteins [69]. Levodopa and dopaminergic agonists are the gold standard of PD treatment, aiming to restore dopamine levels in the basal ganglia [65]. However, these agents fail to inhibit the progression of the neurodegenerative process, their effectiveness diminishes over time, and after prolonged use, patients usually develop motor complications. Although the pathogenesis of PD has not been fully elucidated, both genetic and environmental factors contribute to its development. Mutations in the genes encoding α-synuclein, PTEN Induced Kinase 1 (*PINK1*), leucine-rich repeat kinase 2 (*LRRK2*), and *Parkin* constitute some of the most common genetic causes of PD [69]. Several molecular and cellular mechanisms are implicated in its pathophysiology, including dysregulation of apoptosis, oxidative stress, autophagy and proteasome dysfunction, mitochondrial impairment, neuroinflammation, and dysregulated dopaminergic neurotransmission, while the role of epigenetic regulation is gaining increasing attention [70].

### 5.1. PRMTs and Dopaminergic Neurotransmission in PD

Dopaminergic neuronal death in the SNpc and the subsequent nigrostriatal denervation leads to dopamine depletion in the striatum, which is linked to the motor manifestations of PD. Dysregulation of dopaminergic neurotransmission in the basal ganglia is critically implicated in PD pathophysiology [69]. Dopaminergic agonists, such as pramipexole, rotigotine and ropinirole, are widely used for PD treatment and act by activating dopamine receptors in the basal ganglia. Dopamine receptors are members of the G-protein-coupled receptors (GPCRs) superfamily, and they are subdivided into D1-like (D1Rs, D5Rs) and D2-like receptors (D2Rs, D3Rs, D4Rs) [71]. D1-like receptors activate adenylyl cyclase via Gαs or Gαolf, leading to higher intracellular levels of cyclic adenosine monophosphate (cAMP), while D2-like receptors interact with Gαi and Gαo, inhibit adenylyl cyclase and reduce cAMP. 

It has been demonstrated that PRMT5 methylates D2R, resulting in the enhancement of D2R-mediated dopaminergic signaling [72]. More specifically, PRMT5 induces the D2R methylation in the third intracellular loop, while the mutation of the conserved arginine residues of this region could inhibit the D2R-induced downregulation of the cAMP pathway in human embryonic kidney (HEK) 293T cell cultures [72]. In vivo, PRMT5 upregulated dopaminergic signaling via the DOP-3 receptor (D2-like receptor) in *Caenorhabditis elegans*, which was accompanied by increased dopamine-mediated regulation of locomotor and chemosensory behavior in the nematodes [72]. Functionally, the N-terminal region of the third intracellular loop of D2R is very important since it interacts with various proteins, including Gαi/o, leading to the inhibition of adenylyl cyclase [73]. Methylation of arginine in this region (Arg217, Arg219) removes a hydrogen bond donor and reduces the electrostatic potential, thereby altering its hydrophobicity and size, which may influence the interaction of the N-terminal region with its binding proteins [72,73]. Hence, PRMT5-mediated D2R arginine methylation may upregulate D2R signaling either by directly affecting the interaction site of the receptor with G proteins and subsequently its activity or indirectly by altering its interaction with other regulatory binding proteins, thereby regulating dopaminergic neurotransmission with potential implications in PD pathophysiology and pharmacotherapy, which should be further investigated.

### 5.2. PRMTs and Dopaminergic Cell Apoptosis in PD

PRMTs are critically implicated in cell death. In particular, PRMT1 and PRMT4 enhance oxidative stress-mediated retinal pigment epithelial cell apoptosis in a sirtuin (SIRT1)-dependent and SIRT1-independent manner, respectively [74], and PRMT1 and PRMT5 stimulate ischemia- and hypoxia-induced apoptosis in lung epithelial cells via the phosphorylation of p38 mitogen-activated protein kinase (MAPK) and c-Jun N-terminal kinase (JNK) [75]. However, their role in dopaminergic neuronal death remained, until recently, largely unexplored. 

In this context, in vitro evidence has shown that treatment of dopaminergic neuronal SN4741 cells with 1-Methyl4-phenylpyridinium iodide (MPP+), paraquat and rotenone, three neurotoxins causing dopaminergic cell death, were able to elevate the expression of PRMT1, while PRMT3, PRMT4 and PRMT5 expression was unchanged [76]. PRMT1 activity was also increased, as indicated by the higher levels of intracellular ADMA levels and enhanced dimethylation of histone 4 at arginine 3 (H4R3). Overexpression of PRMT1 could also enhance dopaminergic cell apoptosis, while PRMT1 knockdown acted in a neuroprotective manner. In line with this evidence, the expression of PRMT1 was elevated in the SNpc of 1-methyl-4-phenyl-1,2,3,6-tetrahydropyridine (MPTP)-treated mice, and PRMT1 haploinsufficiency (prmt1 +/−) was correlated with reduced death of dopaminergic neurons in vivo, indicating the potential critical role of PRMT1 in the apoptosis of dopaminergic neurons in PD [76].

Regarding the underlying molecular mechanisms, in the abovementioned study, it was revealed that PRMT1 could regulate the poly (ADP-ribose) Polymerase 1 PARP1-mediated apoptosis-inducing factor (AIF) nuclear translocation [76]. PARP1 constitutes a DNA repair enzyme implicated in the cellular defense mechanism; it acts by cleaving nicotinamide adenine dinucleotide (NAD+) and transferring ADP-ribose to target proteins, such as histones [77]. On the other hand, PARP1 overactivation results in cell death (PARP1-mediated cell death, also known as parthanatos) via the depletion of the energy supplies adenosine triphosphate (ATP) and NAD+, the translocation of AIF to the nucleus and subsequent DNA fragmentation [77]. PARP1 and AIF are already known to play key roles in PD pathophysiology since PARP1 downregulation can inhibit MPTP-mediated neuronal cell death [78], and elevated AIF translocation to the nucleus has been described in the neuronal cells of patients with PD [79]. Hence, it can be suggested that PRMT1 may promote the PARP1-mediated nuclear translocation of AIF, which could contribute to dopaminergic cell death. 

In addition to PARP1-mediated regulation of AIF, PRMT1 may affect dopaminergic cell apoptosis via additional mechanisms, such as the methylation and modulation of signal-regulating kinase 1 (ASK1). More specifically, PRMT1 can methylate ASK1 at arginine residues 78 and 80, leading to the downregulation of the ASK1 pathway [80]. PRMT1-induced ASK1 methylation can suppress the interaction between ASK1 and TRAF2, which upregulates ASK1, and enhances its interaction with thioredoxin, which downregulates ASK1 [80]. ASK1/JNK and ASK1/p38/NF-κB signaling pathways are implicated in the regulation of apoptosis and neuroinflammation in PD [81,82], suggesting that ASK1 may represent another target of PRMT1 with a potential role in PD pathogenesis.

### 5.3. PRMTs and Iron-Induced Oxidative Stress in PD

Oxidative stress-induced neuronal dysfunction is one of the key mechanisms underlying PD pathogenesis. Iron can form reactive oxygen species (ROS) and reactive nitrogen species (RNS), including NO, thereby contributing to DNA damage, oxidation of proteins, lipid peroxidation and, subsequently, cell death. Excessive iron accumulation is observed in the SNpc of patients with PD [83], and iron promotes cell death of SH-SY5Y cells in vitro in a concentration-dependent manner [84]. 

In diverse types of non-neuronal cells, oxidative stress stimulates both increases and reductions in the levels of PRMT1 [84]. ADMA, one of the products of PRMT1-mediated arginine methylation, inhibits the generation of NO by blocking nitric oxide synthases (NOS), and PRMT1 activates Smad1/5. Iron can enhance Smad1/5 phosphorylation, thereby upregulating the expression of hepcidin, the main modulator of iron homeostasis in the brain. Smad1/5 are transcription factors implicated in neurite outgrowth of the dopaminergic neurons of the midbrain through the bone morphogenetic protein (BMP) pathway [85]. Hepcidin overexpression could prevent dopaminergic neuronal loss, protect against mitochondrial impairment, and reduce α-synuclein accumulation in 6-hydroxydopamine (6-OHDA)- and rotenone-induced models of PD in vivo [86]. Hepcidin was also able to suppress the rotenone-induced accumulation of α-synuclein by regulating autophagy in cultured SH-SY5Y cells [87]. Hence, it has been hypothesized that PRMT1 may be involved in the iron-mediated oxidative stress in PD. Indeed, an in vitro study has shown that iron treatment (100 and 500 μM) was associated with lower levels of PRMT1 in SH-SY5Y cell cultures, which was accompanied by higher iNOS and NO levels, mitochondrial impairment and cell death compared to controls [84]. 

In addition, iron treatment at low concentrations (50 μM) was related to increased Smad1/5 phosphorylation, while exposure to higher iron levels (100 and 500 μM) was associated with reduced phosphorylation of Smad1/5, suggesting that Smad1/5 activation might be a response or compensatory mechanism of SH-SY5Y cells to iron-induced oxidative stress. It can be proposed that Smad1/5 activation may upregulate hepcidin levels, resulting in lower iron levels and, finally, lower ROS and RNS levels. Moreover, under the condition of higher iron concentrations, reduced Smad1/5 phosphorylation might be explained by the decreased PRMT1 levels. Collectively, excessive iron accumulation downregulates PRMT1 in SH-SY5Y cell cultures; the iron-mediated PRMT1 reduction levels might also affect Smad1/5 phosphorylation and cell death, although further evidence is needed to test this hypothesis. 

### 5.4. PRMTs and Other Pathophysiological Mechanisms in PD

PRMTs non-histone substrates include a wide variety of proteins that are involved in numerous cellular functions, whose specific role in PD remains to be elucidated [88]. For instance, PRMT6 can methylate the Forkhead box protein O3 (FOXO3) at arginine 188 and 249, resulting in its activation, and PRMT1 can downregulate PRMT6 leading to FOXO3 deactivation [89]. FOXO3 is a transcription factor acting as a homeostasis modulator of several signaling pathways sensitive to various environmental stimuli (e.g., oxidative and metabolic stress, depletion of growth factors), thereby affecting apoptosis, autophagy, inflammation, and mitochondrial function [90]. *FOXO3* gene polymorphisms are also associated with longevity [90]. Exposure to manganese, an environmental risk factor for PD, can also lead to the downregulation of Sirtuin 1 (SIRT1), contributing to autophagy impairment and neuroinflammation via modulating the SIRT1/FOXO3/LC3B pathway [91]. FOXO3a is ectopically localized to Lewy bodies and Lewy neurites in the brain tissue of PD patients [92], suggesting that it might be involved in α-synuclein aggregation. In PD models, human nigral dopaminergic neurons are very sensitive to alterations of FOXO3 activity: FOXO3 constitutive activation results in apoptosis, while inhibition of FOXO3 transcription leads to oxidative damage [93]. On the other hand, FOXO3 lowers the levels of the soluble form of α-synuclein, triggers the formation of α-synuclein aggregates, and contributes to neuronal loss of dopaminergic cells overexpressing α-synuclein in vivo [93]. LRRK2 can also activate FOXO, resulting in apoptosis of dopaminergic neurons in *Drosophila* models [94]. Although further research is needed, it could be, therefore, speculated that PRMT6 and PRMT1 can activate and deactivate FOXO3, respectively, thereby affecting several key mechanisms in PD, such as apoptosis, neuroinflammation, autophagy dysregulation, oxidative damage, and α-synuclein aggregation.

In summary, PRMTs are implicated in some critical mechanisms underlying PD pathophysiology, including dopaminergic neurotransmission, oxidative stress, mitochondrial impairment, and neuronal apoptosis. In particular, PRMT5 can mediate arginine methylation and upregulate D2R dopaminergic signaling. In studies using neurotoxin-induced models of PD, PRMT1 can enhance dopaminergic cell apoptosis, potentially by promoting the overactivation of PARP1, nuclear translocation of AIF and subsequent depletion of energy supplies. On the other hand, iron-mediated oxidative stress-induced downregulation of PRMT1 has been associated with neuronal cell death and mitochondrial impairment. Hence, the molecular effects of PRMT1 seem to be context-specific and depend on the method used to replicate PD pathophysiology in in vitro or in vivo models.

## 6. Role of PRMTs in Huntington’s Disease

Huntington’s disease (HD) presents a genetic neurodegenerative disorder that is inherited in an autosomal dominant manner, attributed to CAG and, subsequently, polyglutamine (polyQ) repeat expansions in the Huntingtin gene (*HTT*) and protein, respectively. HD is clinically characterized by chorea, cognitive impairment and psychiatric manifestations, and there is no disease-modifying treatment currently available. Subtle psychomotor symptoms and signs of HD usually appear years before diagnosis, accounting for the prodromal phase of HD, which is associated with pathophysiological alterations, such as striatal atrophy [95]. Chorea is often the earliest manifestation, although loss of coordination and bradykinesia may be the more disabling motor features. The mean onset age is 40 years, and the duration of the disease is typically 15–20 years after the initiation of motor symptoms [95]. Earlier age at onset is correlated with longer CAG repeat, while environmental and modifying genetic factors also affect the onset age. On the contrary, the rate of disease progression is less dependent on the CAG repeat length [95]. There is no disease-modifying treatment available till now, while symptomatic treatment includes tetrabenazine for chorea and other drugs for psychiatric symptoms [95].

It has been proposed that huntingtin (HTT)-mediated neurotoxicity is caused by a conformational change in the expanded mutant huntingtin (mHTT), resulting in protein misfolding, aggregation, impaired interactions with other proteins, impaired gene transcription, RNA splicing and cell death [96]. However, the molecular and cellular mechanisms implicated in mHTT-related neurotoxicity remain elusive. HTT is mainly implicated in axonal transport, a function essential for neuronal survival. HTT is a scaffold protein for kinesin-1 and dynein motors, thereby affecting both anterograde and retrograde transport of several molecules, including autophagosomes and brain-derived neurotrophic factor (BDNF) [36]. Proteasome dysfunction, dysregulation of autophagy, impaired mitochondrial dynamics, aberrant ROS production, neuroinflammation, impaired synaptic transmission, and excitotoxicity are also implicated in its pathophysiology [95,97,98,99].

### 6.1. Effects of Huntingtin (HTT) on PRMT5 Activity

PRMT5 mediates the symmetrical dimethylation of arginine (sDMA) in histones H2A, H3, and H4 [100]. HTT acts as a scaffolding factor promoting the formation of protein complexes [101], and PRMT5 can interact with HTT, thereby potentially contributing to mHTT-induced neurotoxicity. Methylosome protein 50 (MEP50) forms a complex with PRMT5 and increases its activity by enhancing its interaction with proteins. MEP50 plays a crucial role in substrate recognition and orientation [102]. In particular, PRMT5/MEP50 forms a tetramer of heterodimers with surface negative charge. MEP50 is necessary for the PRMT5-mediated histone methyltransferase activity of H2A and H4 and binds independently to substrates. The catalytic site of PRMT5 is directed towards the cross-dimer paired MEP50. The activity of PRMT5/MEP50 is suppressed and enhanced by the phosphorylation and acetylation of substrates, respectively; the substrates are centered on MPE50, suggesting that MEP50 binds substrates and increases the activity of PRMT5 regulated by substrate post-translational modifications [102]. 

In this context, it has been shown that PRMT5 co-localizes with both HTT and mHTT in the cytoplasm of primary mouse cortical neuronal cells, and HTT interacts with the PRMT5/MEP50 complex [101]. Interestingly, expanded mHTT displayed a higher co-precipitation with this complex in this study, suggesting that mHTT may exhibit an increased affinity for PRMT5. Furthermore, mHTT was associated with an absence of high molecular weight MEP50 complexes, implying that mHTT could potentially influence the multimerization of MEP50 and its capacity to interact with PRMT5. Regarding the functional consequences of PRMT5/HTT interaction, it was demonstrated that HTT could enhance the histone-modifying activity of PRMT5 in vitro, and this effect was less prominent in the case of mHTT [101]. Arginine dimethylation of histones H2A and H4 was also reduced in human brain tissues of HD patients compared to controls [101]. Additionally, co-transfection of PRMT5 and MEP50 in primary neurons could prevent mHTT-induced toxicity, while the inhibition of Jumonji C domain-containing protein 6 (JMJD6), which removes histone dimethyl arginine marks, was associated with increased arginine methylation of histones H2A and H4 and reduced mHTT-induced toxicity [101]. Collectively, PRMT5 deficiency may contribute to mHTT-induced neurotoxicity in HD, and its pharmacological targeting might represent a useful neuroprotective strategy.

### 6.2. PRMTs, Gene Transcription Regulation and RNA Splicing in HD

PRMT5 induces gene silencing via histone dimethylation at the promoters of various genes, such as γ-globin [100]. Since disrupted transcriptional regulation and an association between reduced H3K4me3 occupancy and lower levels of gene expression have been observed in HD [103], the ability of PRMT5 to affect gene transcription has also been investigated in HD models. In this regard, histone arginine methylation (H2A/H4R3Me2s) was reduced in the promoters of the genes encoding BDNF and γ-globin in the human brains of HD patients compared to controls [101]. BDNF gene transcription is decreased in HD, and BDNF reduction has been associated with earlier onset age of the disease and worse motor function in mouse models of HD [104]. Therefore, PRMT5 might affect BDNF gene transcription in HD, thereby contributing to the detrimental consequences of BDNF reduction. However, given the diverse genes whose transcription is affected by PRMT5, more research is needed in this direction.

RNA splicing is also impaired in HD, and mHTT can interact with proteins implicated in RNA processing [96]. Arginine methylation is a post-translational modification of several RNA-binding proteins participating in RNA splicing. PRMT5 mediates the methylation of spliceosomal Sm proteins and the Cajal body marker coilin, which is implicated in RNA splicing. In HD mouse models and brain tissues of HD patients, coilin methylation was decreased [101]. Although still unclear, impaired PRMT5 could contribute to this reduction.

### 6.3. PRMTs and Huntingtin (HTT) Methylation in HD

Post-translational modifications of HTT and mHTT protein, such as phosphorylation, acetylation and sumoylation, affect its function and toxicity [36]. Proteomic analysis has shown that HTT can interact with several PRMTs, including PRMT1, PRMT3, and PRMT5 [96], and HTT can also form a complex with PRMT2 and PRMT6 [36], suggesting that methylation at arginine residues may represent another post-translational modification of HTT. Indeed, a recent study demonstrated that HTT is arginine methylated by PRMT6 at R118, while loss of PRMT6-mediated arginine methylation impairs the interaction of HTT with vesicles, disrupts its scaffolding capacity, reduces anterograde axonal trafficking, and contributes to neuronal cell death [36]. However, no differences were indicated between methylation-defective HTT and kinesin-1 or dynein interaction, suggesting that other molecular mechanisms underlie the effects of arginine methylation of HTT on axonal trafficking. PRMT6 inhibition in cortical neurons and striatal cells enhances mHTT-mediated neurotoxicity and hinders axonal transport, while PRMT6 overexpression can restore these effects, apart from the case of neurons overexpressing an mHTT methylation defective variant [36]. In vivo, PRMT6 overexpression restores axonal transport and neuromuscular junction defects in *Drosophila* models of HD [36]. Importantly, polyQ expanded mHTT does not impair the methylation of mHTT and the mHTT–PRMT6 complex formation in vivo, suggesting that PRMT6 targeting could be a possibly effective therapeutic approach for HD [36]. Therefore, this evidence suggests that PRMT6 inhibition may be implicated in the pathogenesis of HD by affecting axonal trafficking.

In agreement with this evidence, another recent study demonstrated that PRMT4 and PRMT6 interacted with the N-terminal region and methylate HTT and mHTT at multiple specific arginine residues [105]. HTT arginine methylation improved its solubility, thereby possibly affecting its aggregation status. while methylation-null mutations promoted HTT-induced toxicity in vitro [105]. In HD immortalized striatal precursor neurons (ISPNs), mHTT methylation at R200 and R205 in the N-terminal region was reduced, while methylation at R2016 in the C-terminal region was increased [105]. HD ISPNs displayed lower nuclear levels of mHTT and co-localization with PRMT6, which could possibly explain the decreased R200 and R205 methylation mediated by PRMT6 [105]. On the other hand, PRMT4 co-localization with mHTT was observed in the cytoplasm but not in the nucleus, suggesting that the subcellular localization of mHTT may affect its interaction with PRMTs [105]. Furthermore, PRMT4 and PRMT6 overexpression enhanced neuronal survival [105], implying that PRMT4 and PRMT6 targeting should be explored as a possible therapeutic approach against HD. 

Regarding the molecular mechanisms involved, s-adenosylhomocysteine (SAH) can inhibit PRMTs, and s-adenosylhomocysteine hydrolase (SAHH), which hydrolyses SAH to homocysteine and adenosine, is downregulated in the leukocytes of patients with HD and the striatal region of mouse models of HD [106]. Hence, it could be speculated that higher SAH levels may inhibit PRMT6 in HD, thereby suppressing HTT arginine methylation; however, the specific mechanisms of the potential PRMT6 inhibition in HD need to be investigated.

Finally, a recent meta-analysis of transcriptomic profiles has indicated that PRMT3 mRNA levels were significantly increased in the brains of patients with HD compared to controls in the striatal region of R6/2 mice but not in that of YAC128 mice [107]. PRMT3 has a pivotal role in the neuronal development and maturation of dendritic spines in the hippocampus of rats [33]. Given the fact that R6/2 mice display a more rapid onset and progression of the disease compared to YAC128 mice [107], these differences suggest that PRMT3 dysregulation might be observed in the later stages of the disease. Nevertheless, the effects and underlying mechanisms of PRMT3 activity in HD need to be further explored.

Collectively, HTT has been shown to interact with several PRMTs, including PRMT1, PRMT2, PRMT3, PRMT5 and PRMT6, while the subcellular localization of mHTT has been shown to affect its interaction with PRMTs. It seems that HTT can interact with the PRMT5/MEP50 complex and enhance the histone-modifying activity of PRMT5 to a greater extent compared to mHTT, while the PRMT5/MEP50 complex may prevent mHTT-induced neurotoxicity. PRMT5 activity may also affect the expression of the *BDNF* gene, while PRMT6 inhibition can disrupt the interaction of HTT with vesicles, contributing to impaired axonal transport and neuronal cell death. This evidence suggests that HTT and mHTT can affect the activity of PRMTs, while PRMTs can alter the interaction of HTT and mHTT with proteins and subsequently influence their function and mHTT-induced neurotoxicity. The exact underlying mechanisms and functional consequences of this dynamic imbalance remain to be elucidated.

## 7. Role of PRMTs in Spinal Muscular Atrophy (SMA)

Spinal muscular atrophy (SMA) is an autosomal recessive neurodegenerative disorder denoted by progressive loss of lower alpha motor neurons in the anterior horns of the spinal cord, leading to weakness and atrophy of the trunk and limb muscles [108]. SMA is clinically heterogeneous with variable severity: its early-onset type is fatal in infancy, whereas milder types result in severe disability and respiratory difficulties with longer survival in adulthood [108]. SMA is attributed to mutation or deletion of the survival of motor neuron 1 (*SMN1*) gene on chromosome 5, leading to lower amounts of the survival of motor neuron (SMN) protein [108,109]. SMN is also produced by the *SMN2* gene, in which a single nucleotide polymorphism (C/T) of exon 7 transforms a splicing enhancer into a silencer, thereby omitting exon 7 from most SMN2 mRNAs and generating an unstable truncated isoform (SMN17). However, in case of incomplete penetrance, functional full-length SMN can still be generated by some of the SMN2 mRNAs without exon 7 skipping. SMN severity is critically determined by the copy number of *SMN2*. Two revolutionary therapies, gene therapy and the antisense oligonucleotide nusinersen, aiming to enhance the inclusion of exon 7 in the transcripts of *SMN2*, have been recently approved.

### PRMTs, Gene Transcription Regulation and RNA Splicing in SMA

SMN is critically implicated in RNA splicing, a process regulated by PRMT5 [110]. In particular, SMN forms a complex with Gemins 2–8 and Unrip, and the SMN-Gemins complex cooperates with the PRMT5 complex (methylosome), consisting of PRMT5, WD45 and plCln [109,111]. SMN can bind to dimethylated proteins, including Sm proteins, via its Tudor domain. SMN-Gemins and PRMT5 complexes act as chaperons, contributing to the assembly of Sm proteins with small nuclear RNAs (snRNAs) in order to produce small nuclear ribonucleoproteins (snRNPs), the basic constituents of the spliceosome [111,112,113]. In particular, the Sm D2/D1 dimer is associated with pICln, leading to their delivery to the PRMT5 complex. The symmetrical dimethylation of Sm proteins B/B’, D1 and D3 crucially affects their binding SMN Tudor domain [114]. PRMT5 symmetrically methylates arginines of Sm proteins in an ATP-dependent manner, a process considered to increase their interaction with SMN-Gemins complex and finally prevent premature RNA interactions [110,111]. The interaction of SMN with the spliceosome via its Tudor domain is disrupted in motor neurons of SMA patients, leading to decreased Cajal bodies and impaired Cajal body-dependent biogenesis of snRNPs [115]. Several mutations in SMN1 related to SMA inhibit the binding of SMN to Sm proteins [110]. Moreover, a missense mutation in SMN (E134K) located in the Tudor domain can impair an aromatic cage that binds dimethylarginines of the Sm proteins, and the E134K mutated SMN displayed exhibited lower snRNP assembly activity [112]. 

PRMT5 catalyzes the symmetrical arginine dimethylation at R1810 of the carboxy-terminal domain (CTD) of the POLR2A subunit of RNA polymerase II, which is highly involved in gene transcription, chromatin remodeling, and RNA processing [116]. This POLR2A dimethylation recruits SMN via its Tudor domain, and SMN can subsequently interact with senataxin, which resolves R-loops in transcription termination regions [116]. Therefore, it has been proposed that dimethylated POLR2A at R1810 by PRMT5 might affect transcription termination and be implicated in HD pathogenesis. 

Hence, PRMT5 seems to play a crucial role in RNA splicing and gene transcription in SMA. In particular, PRMT5 can mediate arginine methylation of Sm proteins, which may enhance their interaction with SMN, thereby possibly preventing premature RNA interactions. Furthermore, PRMT5 mediates the symmetrical arginine dimethylation of the POLR2A subunit of RNA polymerase II, which may subsequently affect gene transcription and RNA processing. 

## 8. Role of PRMTs in Spinobulbar Muscular Atrophy (SBMA)

Spinal and bulbar muscular atrophy (SBMA), also called Kennedy’s disease, is a neurodegenerative X-linked recessive proteinopathy caused by CAG repeat expansions in exon 1 of the gene encoding the androgen receptor (AR) [117]. Male patients display muscle weakness and atrophy, as well as endocrine and metabolic abnormalities, whereas even homozygous female patients manifest mild or no symptomatology [117]. The onset age of the disease is between 18 and 64 years [118]. Affected males may present with proximal and distal muscle weakness and atrophy, bulbar involvement with dysarthria and dysphagia, cramping, and perioral fasciculations [119]. Gynecomastia, testicular atrophy, impotence, and metabolic changes may also exist, suggesting that SBMA is rather a multisystemic disorder [118,119]. Compared to other motor neuron diseases, such as ALS, the rate of progression of SBMA is slower, with muscle strength decreasing approximately by 2% per year [118].

The mutated AR results in the degeneration of lower alpha motor neurons and skeletal muscle cells mainly via toxic gain-of- but also loss-of-function mechanisms [120]. The nuclear accumulation of mutant AR protein plays a key role in the pathogenesis of the disease, while additional mechanisms, such as DNA binding of AR and inter-domain interactions, are considered to be necessary for pathogenic AR-mediated toxicity [121]. Downstream molecular alterations, such as transcriptional dysregulation, aberrant axonal transport, and mitochondrial impairment, are also involved in SBMA pathophysiology [121,122]. 

### PRMTs and Androgen Receptor (AR) Interaction

AR undergoes several post-translational modifications, such as phosphorylation by Akt, which decreases AR binding to androgens, its transactivation, and acts protectively against neurodegeneration [123]. Without the ligand, AR is mainly localized in the cytosol, while upon its binding with androgens (testosterone and dihydrotestosterone), it translocates into the nucleus, in which it interacts with androgen-responsive elements (AREs) in order to modulate the transcription of androgen-responsive genes. AR acts as a transcription factor and interacts with several transcriptional co-regulators in order to form functional transcription complexes. Interestingly, PRMT6 was shown to be co-localized with mAR in the motor neurons of the anterior horns of the spinal cord in specimens from a patient with SBMA [123]. PRMT6 was demonstrated to interact and co-activate both AR and, to a greater extent, mAR in both neuronal and patient-derived cells [124]. Mechanistically, PRMT6 interacts with AR via LXXLL, its steroid hormone receptor interaction motif, and the AR activating function 2 (AF-2) surface. PRMT6 is necessary for AR transactivation and acts by methylating arginine residues at Akt consensus site motifs, while serine phosphorylation and arginine methylation are mutually exclusive at these sites [124]. Functionally, the PRMT6/AR interaction altered the expression of the cyclin-dependent kinase inhibitor 1 (*p21CIP/WAF1*), sarco(endo)plasmic reticulum Ca^2+^ ATPase 2b (*SERCA2b*), and vascular endothelial growth factor receptor 2 (*VEGFR2*) genes in cells expressing mAR. In this study, the increased PRMT6/mAR interaction was also shown to result in reduced cell viability and enhanced mAR aggregation in models of SBMA in vitro [123]. In vivo, PRMT6 was indicated to modify the SBMA neurodegenerative phenotype in fly models of SBMA [123]. In particular, PRMT6 inhibition hindered the mAR-mediated toxicity in vitro and in vivo, while PRMT6 overexpression promoted this toxicity. Hence, PRMT6-induced arginine methylation is critically implicated in SBMA pathogenesis. 

Lysine-specific demethylase 1 (LSD1) is another transcriptional co-regulator that activates AR, whereas both PRMT6 and LSD1 contain LXXLL, a steroid hormone binding motif [120]. In this context, it has been hypothesized that PRMT6 and LSD1 may synergistically affect the toxic gain-of-function of AR. A recent study demonstrated that PRMT6 and LSD1 are upregulated in the skeletal muscle of male transgenic mouse models of SBMA and, to a lesser extent, in female ones [120]. A higher occupancy of mutated polyQ AR (mAR) was observed at AREs sites of the promoters of PRMT6 and LSD1, suggesting that mAR might enhance PRMT6 transcription [120]. Furthermore, it was indicated that PRMT6 interacts with LSD1 to cooperatively promote AR and, to a greater extent, mAR transactivation, while PRMT6 and LSD1 are necessary for the ligand-mediated AR transcriptional response [120]. Moreover, PRMT6 and LSD1 synergistically enhanced the mAR-induced neurodegenerative phenotype of Drosophila models of SBMA [120]. PRMT6 and LSD1 gene silencing could also rescue the mAR-induced transcriptional dysregulation of several genes. In vivo, LSD1/PRMT6 gene silencing was associated with improved phenotype of mouse models of SBMA, as demonstrated by better motor coordination and muscle strength [120]. In addition, this treatment was related to decreased accumulation of monomeric and high molecular weight AR in the skeletal muscle of the animals in this study. SBMA is also characterized by the upregulation of various genes in skeletal muscles, including muscle associated receptor tyrosine kinase (MUSK), neural cell adhesion molecule (NCAM) and myogenin (MYOG). LSD1/PRMT6 gene silencing could restore the upregulation of these genes in animal models [120]. In summary, it seems that in SBMA, mAR promotes the overexpression of PRMT6 and LSD1, its own transcriptional co-regulators, in skeletal muscles, which in turn promote the function of AR. In addition, a therapeutic strategy to inhibit mAR toxic gain-of-function could be the targeting of these overexpressed co-regulators. 

In addition to PRMT6, AR and mAR have been shown to form a complex with PRMT2 and PRMT7 [123]. Although these two PRMTs are not able to regulate AR or mAR transcriptional activity in this study [123], their role in SBMA needs further investigation. 

Altogether, PRMT6 seems to be necessary for AR transactivation and enhances mAR-induced neurotoxicity, while PRMT6/mAR interaction may promote mAR aggregation. In addition, PRMT6 and LSD1 can synergistically increase mAR-induced neurodegeneration, while LSD1/PRMT6 gene silencing was able to restore the upregulation of *MUSK*, *NCAM* and *MYOG* genes. Therefore, PRMT6 activity may be implicated in SBMA pathophysiology, and PRMT6 inhibition could represent a promising therapeutic strategy.

## 9. Conclusions and Future Perspectives

Collectively, accumulating evidence highlights the crucial involvement of several PRMTs in the pathophysiology of neurodegenerative diseases, including AD, PD, FTD-ALS spectrum, HD, SMA and SBMA. Underlying mechanisms include the regulation of gene transcription and RNA splicing, as well as their implication in various signaling pathways related to oxidative stress responses, apoptosis, neuroinflammation, vacuole degeneration, abnormal protein accumulation and neurotransmission. 

Given the emerging role of PRMTs in the pathophysiology of neurodegenerative diseases, pharmacological targeting of disease specific PRMTs may represent a valuable therapeutic strategy. Pharmacological targeting of enzymes that mediate the post-translational modifications of key proteins represents a promising therapeutic approach for several diseases. Enzymes involved in arginine methylation have already emerged as useful therapeutic targets for cancer. Several PRMTs inhibitors have been developed against various tumors and hematological malignancies, and they are also being tested in clinical trials [125] (Table 2). Among them, JNJ-64619178 is a PRMT5 inhibitor that has been investigated in clinical trials for non-Hodgkin lymphoma and advanced solid tumors [125]. In addition, inhibitors disrupting the PRMT5/MEP50 complex formation have attracted increasing interest [125]. In addition, targeting enzymes able to demethylate arginine may represent another therapeutic strategy aiming to tightly regulate the arginine methylation status of key disease-related proteins. 

Furthermore, targeting the upstream regulators of PRMTs is another potential approach. In some cases, the activity of PRMTs can be modulated by other post-translational modifications, including phosphorylation; for instance, the phosphorylation of PRMT5 at tyrosine by Src kinases or JAK2-V617F reduces its methyltransferase activity [125].

Gene silencing represents a useful therapeutic strategy against neurodegenerative disorders caused by toxic gain-of-function mechanisms. For instance, amyotrophic lateral sclerosis (ALS) due to superoxide dismutase 1 (SOD1) gene mutations can be intrathecally treated with an ASO against SOD1, demonstrating promising clinical effects [126]. In this context, gene silencing of PRMTs via ASO could be a valuable treatment approach against neurodegenerative diseases. As mentioned above, PRMT6 targeting might improve SBMA phenotype, for example, since it is critically involved in the mAR-induced degenerative process in vivo. 

Notably, PRMTs are implicated in various important cellular mechanisms, such as gene transcription regulation, RNA processing and splicing, cell cycle and proliferation, diverse signaling pathways, and DNA repair, among others, via the methylation of a wide variety of substrates [125]. In addition, the activity of PRMTs might differ between tissues and different conditions. Given the fact that PRMTs and arginine methylation are essential elements for homeostasis and physiological cell growth, non-selective PRMTs inhibition may result in detrimental consequences [125]. The seemingly opposing effects of the various PRMTs in the wide spectrum of neurodegenerative diseases described above further highlight their context-specific role and the importance of the different models used in each study. Research on PRMTs and arginine methylation is in its infancy, especially in the case of neurodegenerative diseases, and it is clear that further preclinical evidence is required before the clinical translation of these findings and the development of PRMT targeting treatment approaches. Therefore, in order to develop effective and safe therapeutic strategies, the substrates and downstream pathways of PRMTs need to be elucidated. 

## Figures and Tables

**Table 2:** Examples of available PRMT inhibitors and their targets.

Type of PRMT Inhibitor	Inhibitor Name	PRMT Target
Type I PRMTs	Allantodapsone	PRMT1
	GSK3368715	Type I PRMTs
	AMI-1	Type I PRMTs
	MS023	Type I PRMTs
	SGC707	PRMT3
	MS049	PRMT6
	EPZ020411	PRMT6 and other PRMTs
	SGC6870	PRMT6
Type II PRMTs	EPZ015666	PRMT5
	GSK3326595	PRMT5
	LLY-283	PRMT5
	JNJ-64619178	PRMT5
	PF-06939999	PRMT5
	PRT811	PRMT5
Type III PRMTs	SGC3027	PRMT7
PRMTs (nonselective)	GMS	PRMT8, PRMT6, PRMT5, PRMT1, PRMT3
	DB75	PRMT1, PRMT5, PRMT6
	DS-437	PRMT5, PRMT7

## Data Availability

Not applicable.
